# Low Dose Cadmium Inhibits Proliferation of Human Renal Mesangial Cells via Activation of the JNK Pathway

**DOI:** 10.3390/ijerph13100990

**Published:** 2016-10-07

**Authors:** Xiaocui Chen, Jing Li, Zuowang Cheng, Yinghua Xu, Xia Wang, Xiaorui Li, Dongmei Xu, Carolyn M. Kapron, Ju Liu

**Affiliations:** 1 Medical Research Center, Shandong Provincial Qianfoshan Hospital, Shandong University, 16766 Jingshi Road, Jinan 250014, China; chenxiaocui2015@sina.com (X.C.); xiawang121@sina.com (X.W.); 2 Key Laboratory of Molecular and Nano Probes, Ministry of Education, College of Chemistry, Chemical Engineering and Materials Science, Shandong Normal University, Jinan 250014, China; lijing922015@163.com; 3 Taishan Medical College, Taian 271000, China; zwcheng95@163.com (Z.C.); xuyinghua1990@sina.com (Y.X.); xrlisherry@sina.com (X.L.); 4 Department of Nephrology, Shandong Provincial Qianfoshan Hospital, Shandong University, 16766 Jingshi Road, Jinan 250014, China; xudongmei63@163.com; 5 Department of Biology, Trent University, Peterborough, ON K9L0G2, Canada; ckapron@trentu.ca

**Keywords:** cadmium, renal mesangial cells, JNK pathway, proliferation

## Abstract

Cadmium (Cd) is a heavy metal and environmental pollutant. The kidney is the principal target organ of Cd exposure. Previously, we found that low concentration of Cd damages the integrity of the glomerular filtration barrier. However, little is known about the effects of Cd on renal mesangial cells, which provide structural support for the glomerular capillary loops and regulate intraglomerular blood flow. In this study, human renal mesangial cells (HRMCs) were cultured in the presence of serum and treated with 4 μM Cd. We found that Cd activates the c-Jun N-terminal kinase (JNK) pathway, and increases the protein levels of c-Jun and c-Fos. Cd treatment also induces a decrease in proliferation and an increase in apoptosis of HRMCs, but only the decrease in HRMC proliferation was reversed by pretreatment with SP600125, an inhibitor of the JNK pathway. In addition, Cd does not change the expression of α-smooth muscle actin and platelet-derived growth factor receptor-β, the markers of mesangial cells, or the alignment of the filamentous actin (F-actin) cytoskeleton of HRMCs. Our data indicate that the JNK pathway mediates the inhibitory effects of Cd on HRMC proliferation.

## 1. Introduction

Cadmium (Cd) is an environmental pollutant, which poses a significant risk to human health due to its long biological half-life and accumulation in various target organs [1,2]. For the general population, Cd exposure is mainly from polluted air, food or drinking water [3]. The kidney is one of the primary organs targeted by Cd [4]. Cd accumulation in proximal tubules causes a loss of reabsorptive capacity [5]. In addition, Cd exposure directly damages the glomerulus, resulting in proteinuria [6]. Cd may affect the filtration function of the kidney through its cytotoxic effects, but the molecular mechanisms are not fully understood.

The glomerulus is a network of capillaries surrounded by mesangial cells and podocytes [7]. The glomerular filtration barrier (GFB) is freely permeable to water, as well as small- and mid-sized solutes in plasma, yet maintains considerable size and charges selectivity for proteins and larger molecules [7]. This barrier has three major components: the fenestrated endothelial cells, the glomerular basement membrane (GBM), and the podocytes with their “slit diaphragms” [8]. The renal mesangial cells (RMCs) are smooth muscle-like cells surrounded by pericellular matrix, providing structural support for the glomerular capillary loops. Mesangial cells are also thought to regulate intraglomerular blood flow through contraction and modification of the luminal surface area [9,10]. Therefore, mesangial cells are a prominent cell type for the maintenance of structural and functional integrity of the glomerulus. The mesangial cells are recognized by the expression of specific markers, including α-smooth muscle actin (α-SMA) and platelet-derived growth factor receptor-β (PDGFR-β) [11,12]. Previous studies reported the disruption of the actin cytoskeletal structure of mouse mesangial cells exposed to 10 μM Cd [13]. However, the detailed mechanisms of Cd-induced toxicity on mesangial cells are still unclear.

Mitogen activated protein kinases (MAPKs) are a family of serine/threonine protein kinases that play an important role in connecting extracellular signals to intracellular regulatory proteins [14]. There are three major members: extracellular signal-regulated kinase (Erk1/2), c-Jun N-terminal kinase (JNK), and p38 MAPK. JNK, also called stress-activated protein kinase (SAPK), is activated by stress signals including UV irradiation, H_2_O_2_, osmotic stress, and ischemia-hypoxia [15]. Downstream effectors of the JNK pathway includes c-Jun, c-Fos, and c-Myc transcriptional factors. c-Fos and c-Jun, which form a heterodimer, are the most common members of the activator protein-1 (AP-1) family [15]. Activated JNK translocates to the nucleus and activates c-Jun [16], leading to the formation of AP-1, which regulates the expression levels of many stress-response genes. JNK is involved in cell survival and apoptosis, while Erk1/2 activation is associated with cell proliferation [14]. However, recent studies have indicated that JNK plays a crucial role in cell proliferation for a variety of cell types [16,17]. In addition, JNK activity leads to F-actin remodeling for cell alignment [18].

In the present study, we examined the effects of low dose Cd (4 μM) on human renal mesangial cells (HRMCs). We found that Cd activates the JNK pathway, increasing the protein levels of c-Jun and c-Fos. In addition, Cd induces a decrease in HRMC proliferation and an increase in apoptotic rate, but does not change the alignment of the F-actin cytoskeleton of HRMCs. SP600125, a JNK inhibitor, reverses the Cd-induced decrease in proliferation but not the increase in apoptosis. This study demonstrates a role for the JNK pathway in mediating the effects of Cd on HRMCs.

## 2. Experimental Section

### 2.1. Cell Culture

Human Renal Mesangial Cells (HRMCs) were purchased from ScienCell Research Laboratories (Carlsbad, CA, USA) and cultured in Dulbecco’s modified Eagle’s medium (DMEM) (Corning Inc., Corning, NY, USA) including 10% fetal bovine serum (Lonza, Basel, Switzerland) and 1% antibiotics (100 IU/mL penicillin and 100 μg/mL streptomycin). The cells were maintained in a humidified atmosphere with 5% CO_2_ at 37 °C. CdCl_2_ was purchased from Sigma Aldrich (St. Louis, MO, USA) and dissolved in phosphate buffered saline (PBS) (8.1 mM Na_2_HPO_4_, 1.9 mM NaH_2_PO_4_, 145 mM NaCl, pH 7.4) with a stock concentration of 1 mM. A concentration of 4 μM CdCl_2_ is considered as low dose treatment in this study. SP600125 was purchased from Cell Signaling Technology (Danver, MA, USA) and dissolved in dimethyl sulfoxide with a stock concentration of 1 mM. For experiments with the JNK inhibitor, 10 μM SP600125 was applied to cells 1 h before the treatment of 4 μM Cd.

### 2.2. Western Blotting

HRMCs were washed with PBS and lysed in RIPA buffer (20 mM Tris pH 7.5, 150 mM NaCl, 50 mM NaF, 1% NP40, 0.1% deoxycholate, 0.1% sodium dodecyl sulfate, 1 mM EDTA) supplemented with protease inhibitors aprotonin (1 μg/mL), leupeptin (10 μg/mL) and PMSF (1 mM). Protein concentration was determined using the BCA assay (Bio-Rad Laboratories, Inc., Berkeley, CA, USA). Equal amount of protein (40 μg) for each sample were electrophoresed through a 10% sodium dodecyl sulfate–polyacrylamide gel and then transferred to a PVDF membrane. The membrane was blocked in TBST (20 mM Tris, 150 mM NaCl, 0.1% Tween 20) with 5% non-fat milk proteins at room temperature for 2.5 h before incubation with the primary antibodies overnight at 4 °C. After washing 3 times with TBST, the membrane was incubated with the secondary antibody at room temperature for 2 h. The primary antibodies were rabbit anti-SAPK/JNK (9258), rabbit anti-phospho-SAPK/JNK (4668), rabbit anti-c-Fos (2250), rabbit anti-c-Jun (9165) and rabbit anti-GAPDH (2118) (Cell Signaling Technology, Danvers, MA, USA) and rabbit anti-PCNA (Proteintech, Rosemont, IL, USA). The secondary antibody was HRP-linked goat anti-rabbit IgG antibody (Cell Signaling Technology). The blots were developed with enhanced chemiluminescence reagents (Millipore, Billerica, MA, USA), and the relative intensities of immunoblots were quantified with Image J software (NIH, Bethesda, MD, USA).

### 2.3. Cell Proliferation Assay

HRMC proliferation was evaluated by an MTT assay kit (Cayman Chemical Company, Ann Arbor, MI, USA) following the manufacturer’s protocol. Briefly, the HRMCs were plated with a density of 5×10^3^ cells per well in a 96-well plate and cultured overnight. The next day the cells were treated with 4 μM Cd for 24 h, then MTT solution (10 μL of 5 mg/mL stock) was added to each well and incubated for 4 h. The crystals were solubilized by the addition of 110 μL of formazan dissolving solution, the crystals were solubilized and the colorimetric intensity was analyzed using a 96-well plate reader (Molecular Devices, Sunnyvale, CA, USA) at a wavelength of 490 nm.

### 2.4. Cell Viability Assay

Cell viability was assessed by trypan blue exclusion assay on the HRMCs cultured on the 6 well plates. The wells were washed and incubated in 0.05% trypsin for 2 min at 37 °C. After disaggregation, the single cell suspension of HRMCs was diluted 9:1 with 0.4% trypan blue (Solarbio Science & Technology, Beijing, China) and the percentage of dye-free cells was calculated under a microscope.

### 2.5. Annexin V-FITC/PI Analyses

Apoptosis of HRMCs was determined by Annexin V-FITC and propidium iodide (PI) staining using an assay kit (Neobiosciences, Shenzhen, China) according to the manufacturer’s protocol. Briefly, after treatment, cells were trypsinized, pelleted, washed twice with PBS and resuspended into a single cell suspension. Then, 1×10^6^ cells were stained with Annexin V-FITC (0.025%) for 3 min and PI (20 μg/mL) for 10 min at room temperature in the dark. Positive staining of the cells was detected using a FACSAriaII flow cytometer (BD Biosciences, San Jose, CA, USA) and the data were analyzed using the FACS Diva acquisition and analysis software (BD Biosciences).

### 2.6. Immunofluorescence

HRMCs were grown into monolayer on fibronectin-coated glass chamber slides and were then treated with 4 μM Cd for 24 h. The medium was aspirated, and the monolayers were washed with PBS, fixed with 4% paraformaldehyde, and washed 3 times with PBS for 15 min. Then the cells were permeabilized with 0.1% Triton X-100 for 10 min and washed 3 times with PBS for 15 min. Immunofluorescence was performed by staining with a mouse monoclonal antibody against α-SMAlabellling with fluorescein isothiocyanate (FITC) (Abcam, Cambridge, MA, USA) and a rabbit monoclonal antibody against PDGFR-β (Abcam) at a dilution of 1:50 overnight at 4 °C. The secondary antibody (1:200) for PDGFR-β was Alexia Fluor 546-labeled donkey anti rabbit (Thermo FisherScientific, Waltham, MA, USA) for 2 h at room temperature. The slides were photographed using an Olympus BX51 Imaging System (Olympus Corporation, Tokyo, Japan) with an excitation wavelength of 488 or 546 nm.

### 2.7. Phalloidin-Labelling

HRMCs were allowed to grow to confluency on fibronectin-coated glass chamber slides. After exposure to 4 μM Cd for 24 h, the medium was aspirated and the monolayer was fixed for 5 min in 3.7% formaldehyde solution in PBS. The cells were permeabilized with 0.1% triton X-100 in PBS, washed again in PBS, and stained with 5 μg/mL Phalloidin-Tetramethylrhodamine B isothiocyanate (Sigma Aldrich, St. Louis, MO, USA) in PBS for 1 h at room temperature. After extensive washing, the slides were photographed using a confocal microscope (LSM 880, Zeiss, Oberkochen, Germany).

### 2.8. Statistical Analysis

Statistical significance was assessed using unpaired-sample *t*-tests, *p* < 0.05 was considered significant. Statistical analyses were performed using SPSS 17.0 software (SPSS Inc., Chicago, IL, USA). All experiments were repeated at least three times.

## 3. Results

### 3.1. Low Dose Cd Activates JNK Pathway in HRMCs

**Figure 1:**
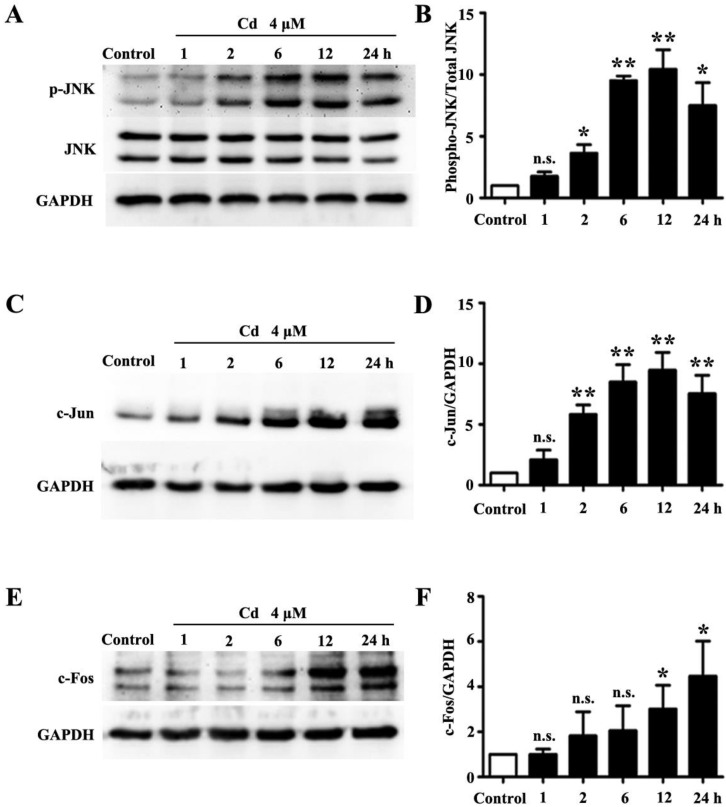
Low dose Cd activates the JNK pathway in human renal mesangial cells (HRMCs). Western blotting analyses from protein samples of HRMCs treated with 4 μM Cd at different time points. The non-treatment samples were used as the control and GAPDH was used as loading control. (**A**) Representative blots of phospho-JNK and total JNK, and GAPDH; (**B**) Densitometry analysis of phospho-JNK/total JNK; (**C**) Representative blots of c-Jun and GAPDH; (**D**) Densitometry analysis of c-Jun/GAPDH; (**E**) Representative blots of c-Fos and GAPDH; and (**F**) Densitometry analysis of c-Fos/GAPDH. n = 3; n.s. non-significant; * *p* < 0.05; ** *p* < 0.01.

Higher concentrations (>10 μM) of Cd induce oxidative stress which consequently activates the JNK pathway in various types of cells [19]. In this study, we examined whether low dose Cd exposure activates the JNK pathway in HRMCs by Western blotting. As shown in Figure 1A,B, the phosphorylated JNK was significantly increased with a peak at 12 h in HRMCs treated with 4 μM Cd, while the total levels of JNK protein and internal control GAPDH remained unchanged. Moreover, protein levels of c-Fos and c-Jun, the downstream effectors of the JNK pathway, were significantly increased (Figure 1C,E).

### 3.2. Low Dose Cd Inhibits Proliferation of HRMC via Activation of JNK Pathway

**Figure 2:**
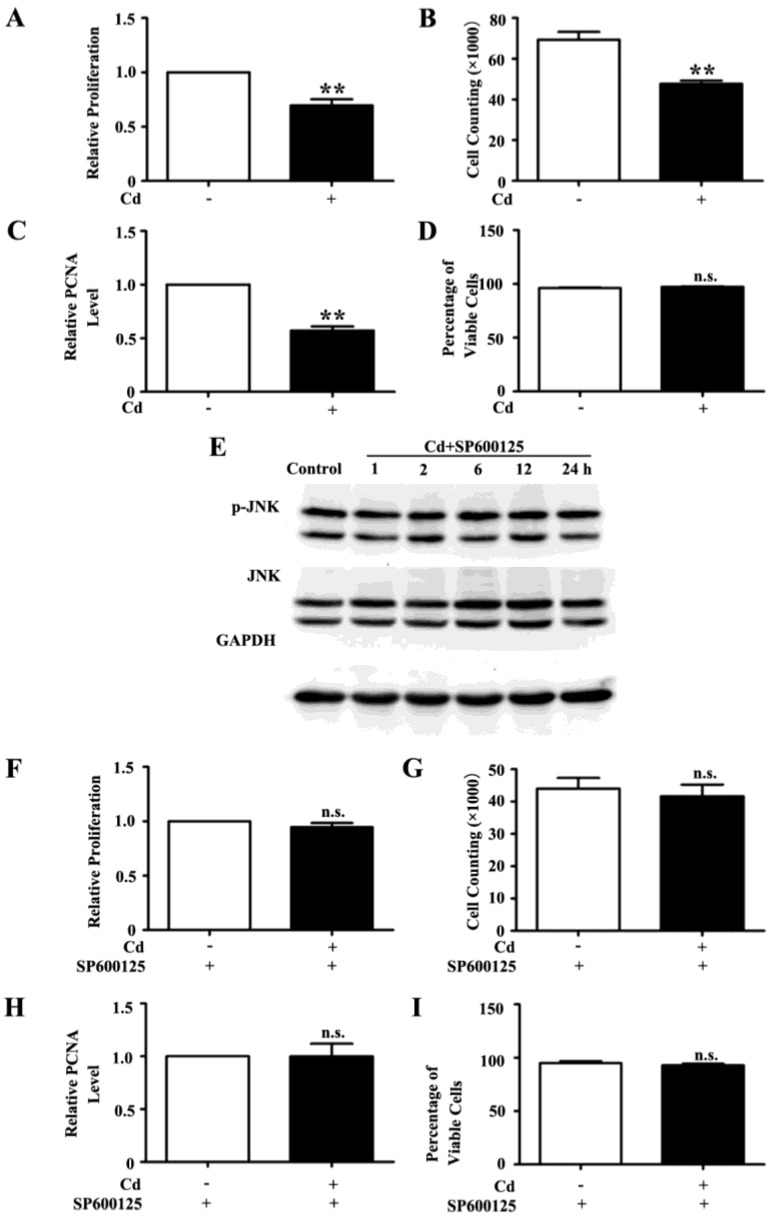
Low dose Cd inhibits proliferation of HRMCs via the activation of JNK pathway: (**A**) MTT assay of HRMCs treated with 4 μM Cd for 24 h. n = 6; ** *p* <0.01; (**B**) Cell counting of HRMCs with or without exposure to 4 μM Cd for 24 h. n = 3, ** *p* < 0.01; (**C**) Densitometry analysis of the immunoblots of PCNA from protein samples of HRMCs with or without exposure to 4 μM Cd for 24 h. n = 3, ** *p* < 0.01; (**D**) Trypan blue exclusion assay of HRMCs treated with 4 μM Cd for 24 h. n = 4; n.s. non-significant; (**E**) Representative blots of phospho-JNK and total JNK from protein samples of HRMCs exposed to 4 μM Cd at different time points with pretreatment with JNK inhibitor SP600125. The cells treated with SP600125 alone were used as control; (**F**) MTT assay of HRMCs treated with 4 μM Cd for 24 h with pretreatment with JNK inhibitor SP600125. n = 6; n.s. non-significant; (**G**) Cell counting of HRMCs treated with SP600125 or a combination of 4 μM Cd and SP600125 for 24 h. n = 3; n.s. non-significant; (**H**) Densitometry analysis of the immunoblots of PCNA from protein samples of HRMCs treated with SP600125 or a combination of 4 μM Cd and SP600125 for 24 h. n = 3; n.s. non-significant; (**I**) Tryplan blue exclusion assay of HRMCs treated with SP600125 or a combination of 4 μM Cd and SP600125 for 24 h. n = 4; n.s. non-significant. The values of the controls were normalized to 1 in A, C, F, and H.

Cd may affect multiple cellular processes including proliferation [20]. The effect of low dose Cd on the proliferation of HRMCs was examined by MTT assay and cell counting. We found that 4 μM Cd decreased the MTT OD reading (*p* < 0.01) and the cell counting of HRMCs (*p* < 0.01) (Figure 2A,B). Proliferating Cell Nuclear Antigen (PCNA), a marker of proliferation [21], was decreased in HRMCs exposed to Cd for 24 h as shown by immunoblots densitometry analysis (1 vs. 0.59 ± 0.02, *p* < 0.01) (Figure 2C). However, trypan blue exclusion assay showed that Cd does not affect viability of the HRMCs (*p* = 0.219) (Figure 2D). SP600125 is a potent, cell-permeable, and selective inhibitor of JNK [22]. Pretreatment with 10 μM SP600125 for 1 h inhibited phosphorylation of JNK in HRMCs exposed to Cd (Figure 2E). We also found that the MTT OD reading, cell counting and PCNA level of HRMCs treated with a combination of SP600125 and Cd was similar to that of treatment with SP600125 alone (*p* = 0.655, *p* = 0.657, *p* = 0.938, respectively) (Figure 2F–H). Upon treatment with SP600125, the cell viability was similar in HRMCs with and without Cd treatment (*p* = 0.447) (Figure 2I). Thus, the JNK pathway mediates the Cd induced decrease in HRMC proliferation. 

### 3.3. Effects of Low Dose Cd Exposure on Apoptosis of HRMCs.

**Figure 3:**
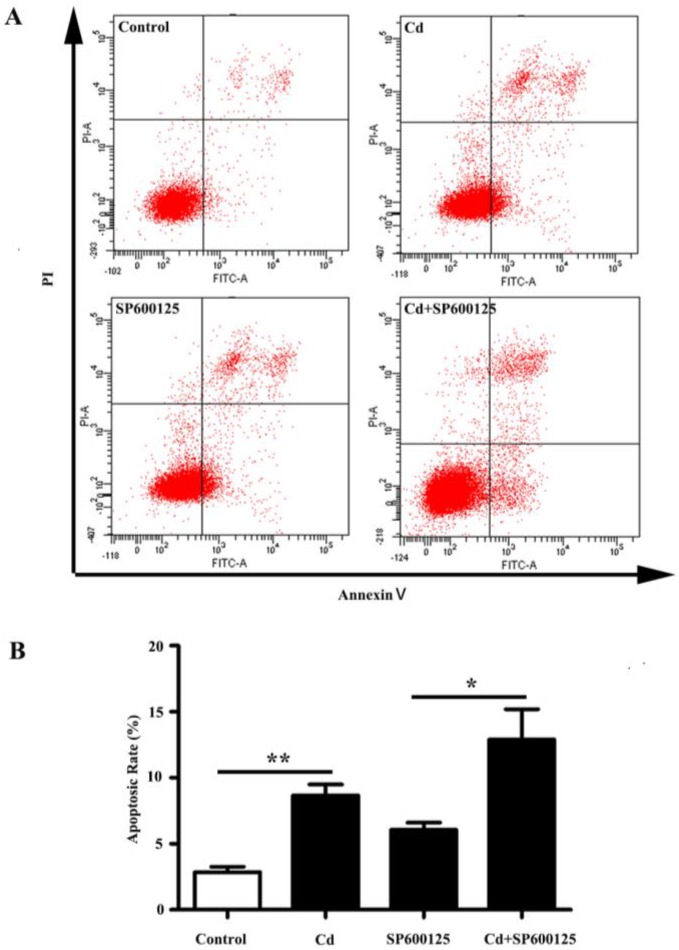
Effects of low dose Cd exposure on apoptosis of HRMCs. (**A**) Representative image of flow cytometry detection with annexin V/PI double staining for HRMCs exposed to Cd, SP600125, or a combination of Cd and SP600125 for 24 h; (**B**) Bar graph of apoptotic rate of HRMCs following flow cytometry. n = 3; ** *p* < 0.01; * *p* < 0.05.

After exposure to 4 μM of Cd for 24 h, apoptosis of HRMCs was examined with Annexin V-FITC/PI double-labeled flow cytometry. The apoptotic rate was calculated as the percentage of the early and late apoptotic cells. As shown in Figure 3A,B, an increase in the apoptotic rate was observed in Cd-treated HRMCs (2.87% ± 0.38% vs. 8.67% ± 0.83%, *p* < 0.01). Pretreatment with SP600125, did not prevent the Cd-induced increase in apoptotic rate of HRMCs (6.07% ± 0.55% vs. 12.90% ± 2.29%, *p* < 0.05). No significant change in the fold increase of the apoptotic rate was observed with Cd or a combination of Cd and SP600125 (3.10 ± 0.34 fold vs. 2.19 ± 0.53 fold, *p* = 0.228). Therefore, 4 μM Cd promotes apoptosis of HRMCs independent of the JNK pathway.

### 3.4. Effects of Low Dose Cd Exposure on theMesangial Cell Markers on HRMCs

**Figure 4:**
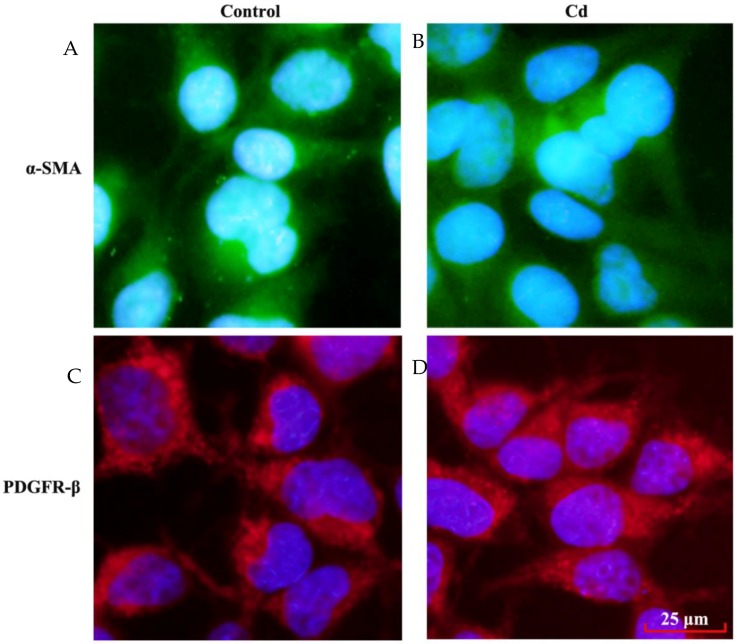
Effects of low dose Cd exposure onα-SMA and PDGFR-β expression in HRMCs. Immunofluoresence staining of α-SMA and PDGFR-β in HRMCsmonolayer treated with PBS (control) and 4 μM Cd for 24 h. (**A**,**B**) Representative images of HRMCs with immunofluoresence staining of α-SMA; (**A**) control; (**B**), Cd treatment; (**C**,**D**) Representative images of HRMCs with immunofluoresence staining of PDGFR-β; (**C**) control; (**D**) Cd treatment.

The mesangial cells are recognized by the expression of a variety of markers, including α-SMA and PDGFR-β [11,12]. In response to extracellular stimuli, mesangial cells may undergo transdifferentiation and lose their specific markers [23]. In this study, the characteristics of HRMCs were examined by immunofloursence staining for α-SMA and PDGFR-β. As shown in Figure 4, expression of α-SMA and PDGFR-β in HRMCs were not altered after 24 h exposure to 4 μM Cd. Therefore, low dose Cd does not induce transdifferentiation of HRMCs.

### 3.5. Effects of Low Dose Cd Exposure on the Actin Cytoskeleton of HRMCs

**Figure 5:**
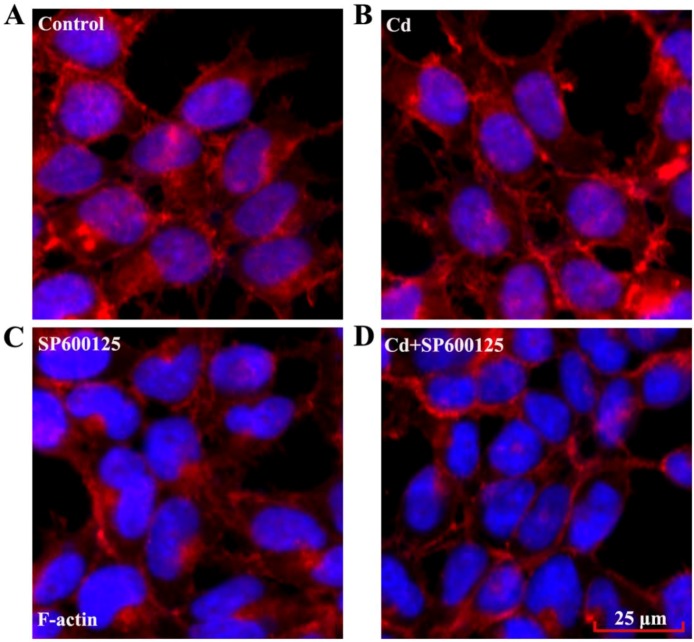
Effects of low dose Cd exposure on F-actin arrangement in HRMCs. Immunofluoresence staining of F-actin with Phalloidin–Tetramethylrhodamine B isothiocyanate in HRMCs monolayer treated with (**A**)PBS (control); (**B**) 4 μM Cd for 24 h; (**C**) 10 μM SP600125 for 24 h; (**D**) 4 μM Cd and 10 μM SP600125 for 24 h.

Cd causes disruption of cytoskeletal components, including actin, in several cell types [24,25]. Cell motility is regulated by the assembly and disassembly of the F-actin cytoskeleton, while F-actin also generates contractile forces to maintain the structure of intercellular contacts [26,27,28]. We examined the F-actin cytoskeleton of mesangial cells by exposing the cells to its interacting molecule phalloidin that was conjugated to a fluorochrome. We found that F-actin arrangement in HRMCs was not disrupted compared to controls, following 24 h exposure to 4 μM Cd (Figure 5A,B). In addition, a combined treatment with SP600125 and Cd did not significantly change the F-actin arrangement in HRMCs compared to the cells with SP600125 alone (Figure 5C,D).

## 4. Discussion

Cd, a widespread pollutant, causes a series of clinical symptoms of kidney diseases in humans [29]. In this study, we explored the effects of low dose Cd on HRMCs, the smooth muscle-like cells surrounding glomerular capillaries. We found that 4 μM Cd decreased proliferation and increased apoptosis of HRMCs, but did not affect the cytoskeleton. Cd treatment increased phosphorylation of JNK and the levels of c-Jun and c-Fos. Inhibition of the JNK pathway prevented the Cd induced decrease in HRMC proliferation. Our study suggests that activation of the JNK pathway mediates the inhibitory effects of Cd on HRMC proliferation.

Cd exposure differentially affects various tissues and cell types depending on exposure dose, route and duration [29,30,31,32]. For example, significant injury has not been found in vascular cells after treatment with a low concentration of Cd [33]. For endothelial cells, Cd induces cell death only at a concentration higher than 10 μM [6]. We have reported that 4 μM Cd does not affect human umbilical vein endothelial cell growth and viability up to 48 h [34]. Low dose Cd does not affect proliferation and apoptosis of human renal glomerular endothelial cells (HRGECs) or podocytes [35,36]. However, kidney proximal tubule cells proliferation was upregulated by low level Cd [37]. In contrast, in our study, we found that low dose Cd exposure inhibits HRMC proliferation. We also determined that 4 μM 24 h Cd exposure induces the apoptosis in HRMCs, which is in accordance with previous findings that Cd induces apoptosis in mouse mesangial cells in a dose- and time-dependent manner [38]. Together, these results suggest that mesangial cells are the earliest target of Cd toxicity in the glomerulus. Renal mesangial cells are critically involved during various types of glomerular injury [39,40]. Loss of mesangial cells disrupts the glomerular capillary network and impairs glomerular ultrafiltration, ultimately resulting in glomerular sclerosis and end-stage renal failure. In addition, the lack of mesangial cells results in the formation of intraglomerular vascular sacs, instead of the normal capillary network [41]. Thus, low dose Cd might damage glomerular function primarily through cytotoxic effects on mesangial cells.

MAPK cascades are evolutionarily conserved intracellular signaling pathways regulating a series of cellular processes [42]. In response to environmental stimuli, members of the MAPK family, including Erk1/2, JNK, and p38 MAPK, are activated by phosphorylation and subsequently activate downstream effectors to promote diverse physiological responses such as proliferation, migration, differentiation and apoptosis. Cd has been shown to activate these three MAPK pathways [43] with differential effects in different cell types. In this study, we found 4 μM Cd treatment activates the JNK pathway and increases c-Jun and c-Fos expression. In addition, the JNK pathway mediates the effect of Cd on proliferation but not apoptosis of HRMCs. Cyclins, c-Myc and p21, the downstream effectors of the JNK pathway, are associated with cell-cycle progression and cell proliferation [44,45]. Consistent with our findings, epoxyeicosatrienoic acids (EETs) increases the expression of cyclin A, cyclin D1 and decreases the expressionof p21 to regulate proliferation via JNK pathway [44]. In addition, SP600125 causes growth arrest by inhibiting c-Myc expression [45]. JNK activation also enhances vascular endothelial growth factor(VEGF)-induced G1/S progression and cell proliferation [46].JNK is activated by stress signals and is known to induce apoptosis in many kinds of cells [16,43,47,48]. However, JNK also regulates PDGF-induced proliferation of human adipose tissue-derived mesenchymal stem cells via the Cdk4-cyclin D1/Rb-dependent pathway [17]. Several studies demonstrate JNK is also involved in Ang-II-induced cell proliferation in cultured HRMCs [14].The JNK pathway plays a vital role in cell growth and survival, however, activation of this pathway does not always result in changes in apoptosis. In our previous study, Cd treatment increases phosphorylated-JNK in HRGECs, but had no effect on HRGEC apoptosis [35]. In addition, Cd activates the protein kinase B (AKT)/mammalian target of rapamycin (mTOR) pathway and directly promotes the mitochondrial apoptotic pathways in several cell types. Taken together, our study suggests that Cd inhibits HRMC proliferation via activation of the JNK pathway but promotes HRMCs apoptosis through other signaling pathways.

Extracellular stimuli may induce phenotype transition in rat mesangial cells [23]. We found 4 μM Cd does not reduce the expression of specific markers of mesangial cells. Thus, the cell type specific properties of mesangial cells are not affected by low dose Cd exposure. F-actin is a major component of the cytoskeleton [49]. It provides structure and scaffolding to the cells, and undergoes dynamic polymerization-depolymerization cycles associated with cell extension and motility [50]. Previous studies reported the effects of divalent metals on F-actin structure in cultured cells and several signaling cascades may be activated with reorganization of the actin cytoskeleton [51]. JNKs are relevant for the integrity of the cytoskeleton and the reception of signals emanating from the cytoskeleton. Yang et al. suggested that phosphorylated JNK associates with actin via α-actinin, an actin-binding protein [52]. JNK is also involved in 4-hydroxy-2-nonenal -mediated actin remodelingin lung microvascular endothelial cells [53]. High dose Cd induces the disruption of F-actin arrangement in rat mesangial cells that are serum-starved prior to Cd treatment [13]. We recently observed that low dose Cd induces hyper-permeability in HRGEC monolayers accompanied by VE-cadherin and β-catenin re-distribution [35]. In our study, 4 μM Cd treatment does not significantly affect F-actin arrangement in HRMCs cultured with 10% serum in the media. In addition, no significant difference in F-actin arrangement is observed in HRMCs treated with SP600125 and Cd compared with SP600125 alone. Therefore, low dose Cd does not affect the F-actin cytoskeleton of HRMCs and the JNK pathway is not involved in F-actin arrangement in HRMCs.

## 5. Conclusions

To summarize, low dose Cd exposure decreases the proliferation of HRMCs and increases apoptosis, without exerting a significant effect on the F-actin cytoskeleton. In addition, Cd activates the JNK pathway, which mediates the Cd induced decrease in HRMC proliferation. Our study helps elucidate the molecular mechanisms underlying the toxic effects of Cd on the glomerulus.
